# Pulmonary Large Cell Neuroendocrine Carcinoma

**DOI:** 10.3389/pore.2022.1610730

**Published:** 2022-10-11

**Authors:** Lan Yang, Ying Fan, Hongyang Lu

**Affiliations:** ^1^ Zhejiang Key Laboratory of Diagnosis and Treatment Technology on Thoracic Oncology (Lung and Esophagus), Cancer Hospital of the University of Chinese Academy of Sciences (Zhejiang Cancer Hospital), Hangzhou, China; ^2^ Department of Thoracic Medical Oncology, Cancer Hospital of the University of Chinese Academy of Sciences (Zhejiang Cancer Hospital), Hangzhou, China; ^3^ Institute of Basic Medicine and Cancer (IBMC), Chinese Academy of Sciences, Hangzhou, China; ^4^ The Second Clinical Medical College, Wenzhou Medical University, Wenzhou, China

**Keywords:** treatment, diagnosis, LCNEC, imaging examination, pathologic features, prognosis

## Abstract

Pulmonary large cell neuroendocrine carcinoma (LCNEC) is a rare subtype of malignant pulmonary tumor. The incidence rate of LCNEC was reported to be 0.3%–3% in lung cancers. Although LCNEC is classified as non-small cell lung cancer (NSCLC), it is more aggressive and malignant than other NSCLC, and its biological behavior is similar to that of small cell lung cancer (SCLC). Most of the LCNEC patients are elderly smoking male and the clinical manifestations are not specific. The imaging manifestations of the tumors are often located in the periphery and the upper lobes, and the enlargement of mediastinal or hilar lymph nodes is common. The diagnosis is mainly based on pathology by the histological features and immunohistochemistry (IHC). Specific neuroendocrine markers such as chromogranin A (CgA), synaptophysin (Syn) and CD56 are usually diffusely positive in LCNEC, and found that insulinoma-associated protein (INSM1) and high rate of Ki-67 are helpful for diagnosis. More differential diagnoses also increase the difficulty of correctly diagnosing LCNEC. The rise of LCNEC molecular typing in recent years may be helpful for diagnosis and subsequent treatment. This review focuses on the epidemiological features, imaging studies, pathology, diagnosis, treatment, and prognosis of LCNEC.

## Introduction

Pulmonary large cell neuroendocrine carcinoma (LCNEC) is a rare primary lung malignancy. It originates from argyrophilic cells in the lung and has the morphology and differentiation characteristics of a neuroendocrine tumor [1, 2]. As a rare, aggressive lung malignant tumor subtype, LCNEC was first described by Travis et al. [3] in 1991, when it was distinguished from other types of neuroendocrine lung tumors, such as typical carcinoid (TC), atypical carcinoid (AC), and small cell lung cancer (SCLC) as an independent neuroendocrine tumor [4]. Later on, in 1999 and 2004, the World Health Organization (WHO) identified LCNEC as a variant of large cell carcinoma (LCC), a type of non–small-cell lung cancer (NSCLC), and one of the four major types of neuroendocrine lung tumors [5]. In 2015, the WHO classified LCNEC again and reclassified it from large cell carcinoma to neuroendocrine carcinoma [6]. In 2021, WHO still defined LCNEC as neuroendocrine carcinoma, while emphasizing its molecular typing, which is expected to assist in diagnosis and subsequent treatment [7]. Although LCNEC is classified as NSCLC in pathological types, it is more aggressive and malignant than other NSCLC, and its biological behavior is similar to that of SCLC [8]. Besides, LCNEC is prone to recurrence and metastasis after surgery and developing resistance to chemotherapy, eventually resulting in a poor prognosis [9, 10]. Currently, as there are only a few studies on LCNEC, there is a lack of accurate epidemiological data and no uniform standard for the treatment plan. Therefore, further research and clinical trials are needed to focus on exploring the treatment and prognosis of LCNEC.

### Epidemiological Characteristics and Clinical Characteristics

Epidemiologically, LCNEC is rare lung cancer, which is currently lacking in the large-scale epidemiological investigation. In general, the overall prevalence of LCNEC represents 0.3%–3% of malignant pulmonary neoplasms, and the incidence of LCNEC in surgical resection lung cancers cases appears to be between 2.1% and 3.5% [11, 12]. Fernandez et al. [12] suggested that the true incidence of lung LCNEC is higher than the data reported in the literature because the cytological specimens of lung LCNEC are relatively difficult to diagnose.

The results of epidemiological characterization and clinical characteristics are summarized in detail in Table 1 [1–22]. As shown in Table 1, a large proportion of the patients were elderly, while the mean age of the patients was 60–70 years. Also, the highest incidence rate was among older adults around the age of 64 years. In terms of the sex ratio, patients with LCNEC were predominantly male. In addition, according to the statistics shown in Table 1, 92.8% of LCNEC patients had a history of smoking, which indicated that smoking might be a risk factor for LCNEC. Moreover, LCNEC often occurs in patients who are heavy smokers [23, 24]. Most LCNEC patients tend to be asymptomatic (tumor is accidentally found during physical examination) and may present with chest pain, cough, hemoptysis, and other symptoms, while night sweats, obstructive pneumonia, and paraneoplastic syndrome rarely occur [13, 21]. The clinical course of LCNEC is more aggressive than that in other NSCLCs, and it grows rapidly with a high mitotic index [8,9]. Due to the high degree of malignancy of LCNEC, distant metastases are often a well-known problem in these patients, which often occurs in the liver, bone, brain, and adrenal gland [14, 25, 26].

**TABLE 1:** The reported clinical characteristics and outcomes of LCNEC.

Ref	No.of cases	Age median (range)	Sex			Stage		Overall survival for patients with LCNEC
			Male	Female	Smoking	Stage I/II	Stage III/IV	5 years OS(%)
[1]	141	66 (38–88)	126	15	139	85	54	40.3
[13]	144	63 (35–80)	117	27	135	102	42	43
[14]	48	63.7 (39–81)	41	7	41	40	8	21
[15]	21	62.7 (NM)	14	7	NM	14	7	47
[16]	49	64(NM)	31	18	43	0	49	21–62
[17]	10	70 (51–79)	9	1	10	10	0	15–57
[18]	72	64 (35–82)	62	10	NM	34	38	32.3
[19]	100	64 (41–86)	54	46	98	71	29	15–57
[20]	45	64(NM)	43	2	42	0	45	27
[21]	83	64.8 (41–89)	73	10	80	70	13	27
[22]	139	61.4 (64–73)	126	13	116	114	25	52

NM, not mention.

### Imaging Examination

Chest computed tomography (CT) is an important and basic examination method for lung cancer detection. Unlike other pulmonary neuroendocrine tumors, in LCNEC patients, tumors are mostly located in the periphery of the lung, while only a very small number of patients with LCNEC present with central tumors [14, 15, 27]. CT performance revealed that 108 out of 131 patients with LCNEC presented as a peripheral tumor [28–32]. Oshiro et al. [29] and Lee et al. [30] analyzed 69 patients, among whom 40 had tumors in the right lung, and the upper lobes were involved in 53 patients. Therefore, LCNEC was considered more common in the right lung than in the left and more common in the upper lobe. Previous studies have also found that the CT findings in LCNEC patients were also characteristic. Akata et al. [31] reviewed the CT findings of 36 LCNEC patients and reported that mediastinal or hilar lymph node enlargement was frequent, and the most common findings included irregular margins, surrounding emphysema, notching, calcification, pleural indentation, and formation of cavities. However, there is still ambiguity in calcification in LCNEC. Akata et al. [31] also reported calcification in 6 (21%) out of 29 patients. In their study, Takamochi et al. [33] found 3 out of 35 LCNEC patients (9%) with calcification, which is inconsistent with the Oshiro et al. view, who argued that the calcification is not common in LCNEC [29]. Chong et al. [32] analyzed positron emission tomography (PET-CT) results of 37 patients with neuroendocrine lung tumors, including 15 cases of LCNEC. In their study, the maximum standardized uptake value (SUV) of carcinoids (mean, 4.0; median, 3.4), LCNECs (mean, 12.0; median, 10.7), and SCLCs (mean, 11.6; median, 11.7) (*p* = 0.006) were significantly different, among which the maximum SUV of LCNECs >13.7 suggested a poor survival period (ROC analysis area under the curve, 0.850; *p* < 0.001). Therefore, PET-CT may be useful in predicting the prognosis of LCNEC.

### Laboratory Examination

Tumor markers have an important role in the diagnosis of malignant tumors. Neuron-specific enolase (NSE), as a specific neuroendocrine marker of pulmonary neuroendocrine tumors, is elevated in LCNEC [18, 34]. Other elevated serum markers have also been found in LCNEC. The expression of serum tumor markers carcinoembryonic antigen (CEA) and pro-gastrin-releasing peptide (proGRP) was elevated in LCNEC [34]. Iyoda et al. [18] analyzed the serum tumor markers of LCNEC and identified lactic dehydrogenase (LDH), tissue polypeptide antigen (TPA), CEA, and NSE as significantly increased, and *α*-fetoprotein (AFP), carbohydrate antigen 199 (CA199), and carbohydrate antigen 125 (CA125) to be elevated in only a few patients. The changes in these tumor markers may be helpful in diagnosing LCNEC, but further clarification is needed.

### Pathology and Immunohistochemistry Examinations

The pathology criteria for LCNEC according to the current WHO classification in 2021 still follows the original criteria including: 1. Neuroendocrine morphology with organoid nesting, palisading, rosette-like structures and granular chromatin; 2. Non-small cell cytological features including large cell size, low nuclear/cytoplasmic ratio, nucleoli, or vesicular chromatin; 3. High mitotic rate >10 mitoses per 2 mm^2^ (average 60–80 mitoses per 2 mm^2^); 4. Positive immunohistochemistry for at least one neuroendocrine marker such as chromograninA (CgA), synaptophysin (Syn), neural cell adhesion molecule 1 (NCAM1/CD56) or NSE, or electron microscopy [6, 7, 35, 36].

The cut surface of LCNEC showed gray-brown or gray-white necrosis [29, 35]. And, the necrosis of LCNEC is common and often represents extensive necrosis [37]. Under the light microscope, the histologic features of LCNEC are large cell size and abundant cytoplasm, the nuclear chromatin is coarsely granular or vesicular, less uniform, and the nuclear to cytoplasmic ratio is low [35]. LCNEC contains a lot of spindle-shaped and polygonal cells, with prominent pleomorphism, mainly in nest-like growth and organ-like growth [37]. LCNEC also includes other structural features, such as fence-like surrounding leaflets, chrysanthemum-like clusters; trabecular-like; rosettes, and organoids, which are also structural features of neuroendocrine tumors [35, 37]. LCNEC is a tumor with a high mitotic rate that exceeds 10 mitoses per 10 high-power fields, yet, the observed rate is usually much higher [37, 38].

According to LCNEC immunohistochemistry (IHC) analysis, specific neuroendocrine markers such as CgA, Syn and CD56 are usually diffusely positive in LCNEC [34, 35, 39]. Based on these conclusions, the IHC results of LCNEC were retrospectively analyzed and the results of IHC are summarized in Table 2. As showed in Table 2, the positive percentage of CD56, cytokeratin 7 (CK7), Syn, CgA, NSE and TTF-1, was found to be 84.9% (440/518), 84.4% (54/64), 74.7% (426/570), 65.3% (278/426), 57.4% (39/68), and 54.8% (153/279). The rates of 34βE12, P63 and CK5/6 were lower than others, the results were 35.9% (23/64), 25.0% (16/64), and 22.2% (47/212), respectively. It is similar to Lyda et al. [45] and Eichhorn et al. [34], the expression rates of CD56, Syn, CgA, and NSE are higher. But in Table 2, the expression rate of CK7 is also higher, which deserves further attention. About 54.8% of LCNEC patients express TTF-1, while a very small number of patients express 34βE12. Sturm et al. [46] thought the expression of TTF-1 and 34βE12 may distinguish LCNEC from basal-like carcinoma, and basal-like carcinoma could be excluded when TTF-1 was expressed, while the presence of 34βE12 expression could exclude LCNEC. Alouthgh Syn, CgA, NSE, and CD56 are highly expressed in LCNEC patients, some studies suggest that these markers do not have sufficient sensitivity or specificity [47, 48]. Studies showed that the expression of insulinoma-associated protein (INSM1) is specifically higher than Syn and CgA in neuroendocrine tumors, which is a nuclear transcription factor. Rooper et al.[49] and Dermawan et al. [50] found that INSM1 was expressed in 91.3% and 75% of LCNEC patients, respectively. These may have some significance for the diagnosis of LCNEC. Sakakibara et al. [48] found that 68% of LCNEC patients (30/44) and 92% of SCLC patients (72/78) expressed INSM1, and also found that INSM1 was positive in 9 of 12 SCLC patients who did not express neuroendocrine phenotypic markers, suggesting that INSM1 was superior to phenotypic markers. Sakakibara et al. [48] also found that SCLC with low INSM1 expression (*n* = 28) had a significantly better prognosis (*p* = 0.040) than the high INSM1 group (*n* = 50), which may also apply to LCNEC patients. Other IHC markers can be found in some studies. Rekhtman et al. [51] found 5 of 39 LCNEC patients with a low-level expression of squamous marker p40, which showed focal staining. Bari et al. [52] sequenced the frozen tissues of 8 cases of SCLC and 8 cases of LCNEC, and found that caudal type homeobox 2 (CDX2), Villin 1 (VIL1) and brain-specific angiogenesis inhibitor 3 (BAI3) were differentially expressed in the two tumors. And it was found by IHC that when CDX2 combined with VIL1 for the diagnosis of LCNEC, its sensitivity and specificity were as high as 81%. These markers may help distinguish LCNEC. Saunders et al. [53] reported that the expression rate of Delta-like protein 3 (DLL3) in LCNEC patients detected by IHC accounted for 65%. However, Ogawa et al. [54] found that only 26 of 70 LCNEC patients had positive DLL3 expression, and found that the five-year OS after adjuvant therapy for patients with DLL3-negative tumors was 90.0%, compared with 26.9% for those without adjuvant therapy (*p* < 0.01). Achaete-scute homolog-like 1 (ASCL1) is a highly specific IHC marker that is only expressed in lung neuroendocrine carcinoma and carcinoids [55]. It is emerged as an important transcriptional marker for SCLC, which may also be applied in LCNEC. Many studies have shown that p16 is positive in LCNEC, and the IHC expression rate is approximately 57%–77.5%, which is lower than that in SCLC, but overall studies suggest that retinoblastoma (RB1) positive, cyclin D1 positive, and p16 negative patterns in IHC are more common in LCNEC [56–58]. Therefore, whether the expression of p16 in IHC can further assist in the diagnosis of LCNEC still needs more exploration.

**TABLE 2:** The frequency of immunohistochemical results of LCNEC.

Ref	IHC results (no. Of patients)^a^								
	CD56	CK7	Syn	CgA	NSE	TTF-1	34βE12	P63	CK5/6
[21]	77/83	NM	44/83	54/83	NM	NM	NM	NM	NM
[34]	49/86	NM	46/81	35/61	39/68	NM	NM	NM	NM
[40]	NM	54/64	55/64	44/64	NM	42/64	23/64	16/64	29/64
[41]	79/86	NM	77/85	72/87	NM	NM	NM	NM	NM
[42]	198/216	NM	176/210	46/84	NM	111/215	NM	NM	18/148
[43]	23/25	NM	10/25	11/25	NM	NM	NM	NM	NM
[44]	14/22	NM	18/22	16/22	NM	NM	NM	NM	NM
Total	440/518	54/64	426/570	278/426	39/68	153/279	23/64	16/64	47/212
Rate	84.9%	84.4%	74.7%	65.3%	57.4%	54.8%	35.9%	25.0%	22.2%

NM, not mention.

^a^ IHC results: Patients who express the biomarker/all patients who tested IHC in the reference (positive frequency).

Ki-67 has an essential role in the control and timing of cell proliferation and can evaluate proliferative activity in different tumor cells [59–61]. Right et al. [60] found that Ki-67 was the lowest in TC patients, followed by AC patients, while it was similar in LCNEC and SCLC patients, in both groups being higher than in TC and AC patients. This suggests that the Ki-67 proliferation index can be used to better distinguish high-grade neuroendocrine tumors from low-grade neuroendocrine tumors [62, 63]. In other words, the Ki-67 is helpful in the diagnosis of LCNEC [59].

### Molecular Characteristics

The gene mutations of LCNEC are sorted out in Table 3 [20, 51, 64–68]. As shown in Table 3, the rate of epidermal growth factor receptor (EGFR) mutation in LCNEC is 11.9% (7/59). And the mutations of tumor protein p53 (TP53), RB1, serine/threonine kinase 11 (STK11), kelch like ECH associated protein 1 (KEAP1) and kirsten rat sarcoma viral oncogene homologue (KRAS) also occurred in LCNEC. The mutation frequency was 82.3%, 39.1%, 20.9%, 18.2%, and 12.7%, respectively. The TP53 mutations mainly affected the functionally critical DNA-binding domain, most of RB1 mutations and many STK11 and KEAP1 mutations are frameshift and nonsense mutations. Also KRAS mutations are predominantly missense mutations [64, 68]. And George et al. [68] found some specific gene changes, including a disintegrin and metalloproteinase with thrombospondin motifs 12 (ADAMTS12) (20%), ADAMTS2 (15%) and growth arrest-specific 7 (GAS7) (12%).

**TABLE 3:** The gene mutations in LCNEC.

Ref	Gene mutation					
	TP53	RB1	KEAP1	STK11	KRAS	EGFR
[20]	NM	NM	NM	NM	NM	0/7
[51]	35/45	17/45	14/45	11/45	10/45	NM
[64]	67/79	37/79	14/79	8/79	NM	NM
[65]	24/36	7/36	5/36	3/39	4/39	5/36
[66]	NM	NM	NM	NM	NM	1/3
[67]	NM	NM	NM	NM	0/13	1/13
[68]	55/60	25/60	13/60	18/60	6/60	NM
Total	181/220	86/220	46/220	40/157	20/220	7/59
Rate	82.3%	39.1%	20.9%	18.2%	12.7%	11.9%

NM, not mention.

With the continuous exploration of genetic testing. Rekhtman et al. [51] performed next-generation sequence (NGS) on 45 LCNEC patients and found that LCNEC patients can be divided into three types according to genes. SCLC-like tumors with mutations or deletions in TP53 and RB1, NSCLC-like tumors were classified with KRAS and STK11/KEAP1 mutations, lacking TP53 and RB1 coalteration. And carcinoid-like tumors, which have MEN1 mutations but tumor mutational burden are lower than SCLC-like tumors and NSCLC-like tumors. Subsequently, George et al. [68] further confirmed the characteristics of each subtype. And in 2021, the WHO emphasized the molecular typing of LCNEC, and it is more and more important to consciously carry out LCNEC, which can provide ideas for subsequent treatment.

### Diagnosis and Differential Diagnosis

Due to the lack of specific clinical manifestations of LCNEC, pathological diagnosis is crucial. Similarly, assessment of mitotic rate are important for diagnosis. However, it is difficult to diagnose LCNEC on small biopsies or cytology, direct diagnosis of LCNEC is only capable on surgery specimen. It is worth noting that when LCNEC is associated with other lung cancer components such as squamous cell carcinoma, adenocarcinoma, giant cell carcinoma and/or spindle cell carcinoma, it is classified as combined LCNEC, but if SCLC components are present, it is classified as combined SCLC. In 2021, the WHO proposed that the molecular typing of LCNEC is helpful to diagnose, and also has a certain guiding role for subsequent treatment [7].

The main differential diagnosis of LCNEC includes SCLC and other types of NSCLC. As a high-grade neuroendocrine carcinoma, SCLC and LCNEC have similar IHC characteristics. The two are mainly differentiated by cytological manifestations, including cell size, abundant cytoplasm and obvious nucleoli. Meanwhile, more than 95% SCLC has complete loss of RB1 expression, and LCNEC has about 50%. Therefore, normal RB1 expression is more likely to be diagnosed as LCNEC when the differential diagnosis is difficult [69]. LCNEC also needs to be differentiated from atypical carcinoids, mainly by cytological features and the nature of necrosis. The degree of cellular atypia and necrosis is far less than that of LCNEC, and the Ki-67 index is mostly less than 20% [70]. Basal-like squamous cell carcinoma may exhibit similar cytological features to LCNEC, but p40 is the main marker to distinguish them (basal-like squamous cell carcinoma is mainly diffusely positive, while a small proportion of LCNEC with p40 positive showed only focal staining) [51, 71]. A SMARCA4-deficient undifferentiated thoracic tumor presents as undifferentiated round cells or rhabdoid cells with diffuse positivity for some synaptophysin, extensive necrosis, high mitosis, and high Ki-67 rates, therefore need to be differentiated from LCNEC. It lacks neuroendocrine features, lacks claudin 4 (epithelial adhesion molecule) expression and shows low or absent keratin immunostaining. In addition, SMARCA4-deficient undifferentiated thoracic tumor lacks SMARCA4 [72, 73].

In addition to the main identifications above, LCNECs also need to be identified from other tumors of origin. For example, ductal carcinoma of the breast and prostate cancer can exhibit a prominent endocrine morphology (relatively single cytology and nested structures) after metastasizing into the lung. In addition, rare monomorphic high-grade tumors may develop in the thoracic cavity in younger patients and/or never smokers, possibly similar to LCNECs, including NUT carcinomas, EBV-associated carcinomas (which may lack the striking features typical of lymphoepithelioma-like carcinomas lymphatic infiltration) and round cell sarcoma (CIC rearrangement or even Ewing, typical synaptophysin expression), need to be identified based on clinical features, imaging, and so on [74].

### Treatment

At present, there is still no standard treatment plan for LCNEC. The treatment strategy of LCNEC is based on a multidisciplinary combination of surgical resection, chemotherapy, radiotherapy, immunotherapy and targeted therapy, and platinum-based chemotherapy plays an important role in LCNEC patients [10, 62].

For LCNEC patients, surgery is not only applicable to stage I patients but also for patients in stage II/III [63]. At present, the commonly used surgical methods for LCNEC include local resection, lobectomy, bilobectomy, sleeve lobectomy, and pneumonectomy [11, 20, 75]. Systematic lymph node dissection is also required during the operation [76]. Zacharia et al. [15] showed that lobectomy or pneumonectomy are the better choices for LCNEC patients in the early stages, as they may improve survival in the absence of lymph node metastases at mediastinal sampling. A retrospective study by Iyoda et al. [76] showed poor outcomes in LCNEC patients after wedge resection and segmental resection, so lobectomy or pneumonectomy was recommended. Still, the recurrence rate of local and distant metastases in LCNEC patients is very high, and LCNEC patients who receive adjuvant therapy after early surgery have a significantly longer time before the tumor recurrence than patients who only receive surgery [17, 72]. In other words, surgery alone is not sufficient, and LCNEC patients should also receive adjuvant chemotherapy or multimodal therapy [10, 19, 77].

The prognosis of LCNEC patients is very poor even in the early stages, and distant recurrence after resection is very common in patients with LCNEC [10, 29, 63]. Therefore, chemotherapy is important for LCNEC patients as a treatment option. Although LCNEC is categorized as NSCLC, the biological behaviors of LCNEC tumors are similar to those of SCLC, so the choice of chemotherapy for LCNEC patients remains controversial [62]. A retrospective study, which included a total of 100 prospective LCNEC cases conducted by Sarkaria et al. [19] revealed that among 25 patients receiving adjuvant therapy and 20 who received a platinum-based regimen, the 5-year overall survival (OS) was 37% for patients who did not receive platinum-based chemotherapy and 51% for patients who did. Sun et al. [20] showed that the response rate of LCNEC to platinum-based chemotherapy was 60%, and that of non-platinum-based chemotherapy was 11%. The chemotherapy regimens and effects of LCNEC are listed in Table 4 [20, 21, 78, 79]. As shown in Table 4, the response rate of LCNEC patients treated with platinum plus etoposide was considerable. Tokito et al. [80] reported that in 10 patients treated with platinum plus irinotecan or platinum plus etoposide, the response rate was 70%. In addition, in their phase II study, Niho et al. [81] analyzed 30 cases of LCNEC treated with irinotecan and cisplatin, finding a response rate of 46.7%. Kenmotsu et al. [82] reported that nedaplatin plus irinotecan was effective and safe for patients with LCNEC. Scholars are also exploring new chemotherapy for the treatment of LCNEC. As shown in Table 4, Yoshida et al. [79] found that amirubicin as the second-line treatment for LCNEC patients was effective, achieving the response rate of 27.7% and considerable survival of patients with LCNEC. The 2021 NCCN guidelines recommend multiple chemotherapy regimens for locally unresectable or metastatic lung neuroendocrine cancer (including LCNEC), noting that second or later line efficacy is very limited [83]. However, Derks et al. [64] proposed LCNEC molecular classification and showed that LCNEC with wild-type RB1 or RB1 mutated is more likely to be treated with chemotherapy for NSCLC.

**TABLE 4:** The chemotherapy regimen of LCNEC.

AVB	Treatment period	Treatment plan	No.of cases	ORR (%)	mPFS	mOS
[20]	First-line	platinum + etoposide	11	73%	6.1 month	16.5 months
	First-line	platinum + taxanes/gemcitabine/pemetrexed/vinorelbine	34	50%	4.9 months	9.2 months
[21]	First-line	platinum + etoposide	12	50%	NM	44 months
	First-line	platinum + taxanes/gemcitabine or gemcitabine alone	15	0%	NM	21 month
[78]	First-line	platinum + etoposide/irinotecan	13	59.1%; (55.6% with irinotecan; 71.4% with paclitaxel)	4.1 month	10.3 months (10.3 months with paclitaxel or irinotecan)
	First-line	platinum + paclitaxel/vinorelbine/docetaxe or paclitaxel alone	9			
[79]	Second-line	amrubicin	18	27.7%	3.1 month	5.1 month

Ref, reference; LCNEC, large cell neuroendocrine carcinoma; ORR, objective response rate; mPFS, median progression-free survival; mOS, median overall survival.

There are few studies on radiotherapy for LCNEC patients, and the effect is still unclear. Sakaria et al. [19] suggested that radiotherapy should be considered in advanced LCNEC patients or those who cannot tolerate surgery and chemotherapy. Rieber et al. [84] suggested that for LCNEC patients, radiotherapy in accordance with the guidelines for NSCLC was a reasonable approach, as was adjuvant radiotherapy for LCNEC patients for whom it is impossible to completely remove the tumor and who do not respond well to chemotherapy. Jiang et al. [85] reported on 1619 LCNEC patients with stage I-III, where only about 65 LCNEC patients underwent radiotherapy, 167 LCNEC patients underwent surgery plus radiotherapy, and 138 underwent postoperative radiation therapy. They found that OS in stage I and II LCNEC patients was not improved by radiotherapy (stage I: OS *p* = 0.719; stage II: OS *p* = 0.136). Nevertheless, the OS in stage III patients was significantly improved by radiotherapy (*p* < 0.001). In a study by Prelaj et al. [86], which included 28 patients with stage III-IV LCNEC who received chest radiotherapy and prophylactic cranial irradiation (PCI), it was found that the median progression-free surviva (mPFS) and mOS in patients who received PCI was longer than in the patients who did not receive PCI (mPFS: 20.5 vs. 6.4 months, *p* = 0.09; mOS: 33.4 vs. 8.6 months, *p* = 0.05). And the mOS and mPFS of the patients who received chest radiotherapy are longer (mPFS: 12.5 vs. 5 months, *p* = 0.02; mOS: 28.3 vs. 5 months, *p* = 0.004). Also Iyoda et al. [62, 76] reported that LCNEC patients with brain metastases had a partial reaction to radiotherapy, suggesting that radiotherapy may be effective for LCNEC patients. Rieber et al. [84] suggested brain radiotherapy as the first choice for LCNEC patients with brain metastases. In short, patients with early-stage LCNEC may not benefit from radiotherapy, while patients with advanced LCNEC may benefit, but more studies are needed to confirm.

The gene mutations of LCNEC are sorted out in Table 3. As shown in Table 3, the rate of epidermal growth factor receptor (EGFR) mutation in LCNEC patients was 11.9% (7/59). Some scholars believe that EGFR tyrosine kinase inhibitors (EGFR-TKI) are not likely to present an effective therapy for LCNEC patients [66, 68]. A case report introduced a patient with metastatic LCNEC harboring EGFR-activating mutation who received the therapy with erlotinib and did not respond to erlotinib at its usual dose, after which he died with a survival of 4 months [66]. However, De Pas et al. [87] found that LCNEC patients with EGFR mutation can be successfully treated with EGFR-TKI, with a time to progression of more than six months. Therefore, more studies are needed to determine whether EGFR-TKI is effective in LCNEC patients. Some studies showed a few of LCNEC patients with anaplastic lymphoma kinase (ALK) rearrangement mutation, and the efficacy of ALK inhibitors in LCNEC patients is still controversial [88, 89]. An analysis of 13 patients with LCNEC, which was conducted by Iyoda et al. [67], revealed that the IHC expression of c-KIT, HER2, and VEGF was 76.9%, 30.8%, and 100%, thus suggesting that anti-VEGF and anti-c-KIT agents may be effective in LCNEC therapy and that HER-2 expression may benefit from the treatment with trastuzumab. Currently, other targeted therapies are also being developed. The mTOR signaling pathway inhibitor everolimus is at the moment mainly used for the treatment of pulmonary neuroendocrine tumors [90]. In a prospective study conducted by Christopoulos et al. [91] that included 49 patients with metastatic LCNEC diagnosed and treated with everolimus in combination with chemotherapy, the mOS of 9.9 months was better compared to the general stage IV LCNEC patients whose mOS was only 4 months in general and 7.7 months after chemotherapy. These results showed that everolimus in combination with chemotherapy is a well-tolerated and effective first-line treatment for patients with metastatic LCNEC. Odate et al. [92] also found that the expression of the tropomyosin-related kinase (Trk) and brain-derived neurotrophic factor (BDNF) in LCNEC patients were higher than that in the control SCLC group, suggesting that BDNF/TrkB pathway may be a potential therapeutic target for LCNEC. According to an analysis of 10 patients with LCNEC that was conducted by Filosso et al. [93], the efficacy of octreotide as adjuvant therapy in LCNEC showed that 9 of these patients (90%) were alive and free of disease. Yokouchit et al. [94] reported a case of intravitreal injection of bevacizumab in the treatment of LCNEC with iris tumor metastasis, in which the tumor disappeared after 1 month of treatment.

Immune checkpoint inhibitors (ICIs) of programmed cell death protein 1 (PD-1) and its ligand (PD-L1) are a standard treatment option for advanced NSCLC and SCLC [95–97]. There are few studies related to immunotherapy in LCNEC, and whether ICIs can bring benefits to LCNEC patients still needs more exploration. Tsumoka et al. [96] analyzed 106 LCNEC patients and found that 10.4% expressed PD-L1. Wang et al. [97] analyzed an LCNEC patient whose PD-L1 protein expression was negative, but the patient had a strong response to pembrolizumab, which brought new possibilities for the prognosis of LCNEC patients. In a retrospective study, 11 out of 37 LCNEC patients were PD-L1 positive. These positive patients were treated with nivolumab or pembrolizumab, and 60% of patients achieved partial remission [98]. In their analysis of 125 patients with advanced LCNEC, Dudnik et al. [99] divided patients into two groups, group A (patients who received ICI, *n* = 41) and group B (patients who did not receive ICI, *n* = 84). Their results revealed that ICIs could indeed improve the quality of life of LCNEC patients and the mOS of patients receiving ICIs treatment was significantly longer than that of patients who did not receive ICIs treatment (12.4 months vs. 6.0 months). The ICIs may also be an effective treatment option for LCNEC.

### Prognosis

LCNEC is a highly malignant tumor with a poor prognosis that is often characterized by distant recurrence after resection, even in early stages, which is why early diagnosis is crucial for prognosis [64]. Derks et al. [100] reported on the prognosis of 952 LCNEC patients, revealing that the multivariate-adjusted OS in LCNEC patients did not significantly differ from SCLC patients but was worse than in other NSCLC patients. Even LCNEC patients with pathological stage I showed a low 5-year survival rate of about 27%–67% [76]. At the same time, patients with LCNEC are often associated with recurrence after surgery. According to a previous study, in 63.9%–82% of LCNEC patients, recurrence appeared within 1 year, and 91% of LCNEC patients experienced recurrence within 2 years after surgery. The common recurrence sites were mediastinum and supraclavicular lymph nodes [17, 22].

Many studies have suggested that the prognostic factors of LCNEC are related to age, gender, treatment, tumor size, metastasis, number of positive lymph nodes, and tumor stage. However, there were no reliable predictors [6, 13, 21, 22, 68, 76]. Veronesi et al. [13] included 70 LCNEC patients with stage I who received and did not receive chemotherapy, found the 3-year survival of 100% and 58% (*p* = 0.077), respectively. The results of their multivariate analysis also suggested that age >64 years old in LCNEC patients was an independent prognostic factor of poor outcome (age >64 vs. age ≤64, *p* = 0.057). Rossi et al. [21] assessed 83 patients with LCNEC and found that adjuvant chemotherapy was effective and significantly improved the survival of patients with LCNEC (p < 0.0001). Rossi et al. [21] also found that tumor stage and size (≥3 cm) were significantly correlated with survival. The mOS times were 24, 23, and 10 months for patients with LCNEC in stages I, II, and III, respectively (*p* = 0.0394), and the mOS times were respectively 24 and 13 months for patients with LCNEC <3 cm and > 3 cm (*p* = 0.0039), respectively. In addition, Morise et al. [101] found that LCNEC patients positive for aldehyde dehydrogenase 1 (ALDH1) in IHC had significantly worse recurrence-free survival (RFS) and OS rates than those with ALDH1 negative (5-year RFS: 39% vs. 67%, *p* = 0.009; 5-year OS: 50% vs. 79%, *p* = 0.021). Their multivariate analysis also revealed that positive ALDH1 expression was an independent adverse prognostic factor. Okui et al. [102] retrospectively reviewed 26 patients with LCNEC who underwent complete resection and showed that the preoperative neutrophil-lymphocyte ratios (NLR) in the peripheral blood was an independent prognostic factor for overall survival in these LCNEC patients (hazard ratio 8.559, *p* = 0.011), while the preoperative NLR was inversely correlated with post-resection survival rates in patients with LCNEC. Christopoulos et al. [103] performed that untreated LCNEC patients in stage IV had significant T-cell repertoire alterations, more pronounced T-cell repertoire alterations and higher blood lymphocyte counts at diagnosis were associated with a longer overall survival (441 vs. 157 days in median, *p* = 0.019), a higher degree of T-cell repertoire normalization after 3 months of therapy with more favourable prognosis (median overall survival 617 vs. 316 days, *p* = 0.036). Li et al. [104] analyzed tumor tissues and corresponding normal tissues from 37 surgically resected LCNEC patients. The results showed that there is no significant association observed between tumor mutation burden (TMB) and PD-L1 expression or CD8^+^T cells infiltration by multiplex immunohistochemistry, and TMB was not significantly associated with prognosis in LCNEC patients. But multivariate analysis showed that high interstitial CD8^+^T cell infiltration was an independent favorable factor for disease-free survival (*p* = 0.030). Furthermore, this study using whole exome sequencing found that TP53 mutation may have favorable OS (*p* = 0.073) and the OS of LCNEC patients who have KEAP1 mutation was shorter (*p* = 0.044).

## Conclusion

LCNEC is a rare lung tumor that is behaviorally aggressive relative to other NSCLC. As a type of lung neuroendocrine carcinoma, the incidence of LCNEC is low and LCNEC mostly occured in elderly male who have a long history of smoking, with non-specific clinical manifestations. The imaging examination of LCNEC showed that tumors are mostly located in the periphery of the lung and the upper lobe of the right lung, usually presenting with lobular signs, irregular margins and spiculation. The diagnosis of LCNEC is mainly based on pathology, but it is difficult to diagnose LCNEC by small biopsy or cytology. Correct differential diagnosis is very important. There is no consensus on the treatment of LCNEC patients. The main treatment strategy for early LCNEC patients is a multidisciplinary combination of surgical resection, supplemented by chemotherapy, radiotherapy, targeted therapy and immunotherapy. And for patients who have lost the chance of surgery, chemotherapy is the main treatment. But the chemotherapy regimen at present is not unified and SCLC-based regimens such as platinum plus etoposide or platinum plus irinotecan might be effective. The expression of PD-L1 in some LCNEC patients may be a choice in immunotherapy, which need further clinical investigation. Clear molecular typing of LCNEC may provide direction for treatment. Although the rate of EGFR mutation in LCNEC patients is low, the effectiveness of EGFR-TKI need to be further studied. With the continuous advancement of molecular research, the changes of gene profiles in lung LCNEC suggest that the three molecular types of LCNEC overlap with SCLC, NSCLC, and lung TC, but a large amount of data is still needed for verification. These genes may become molecular markers for the diagnosis of pulmonary LCNEC. On the basis of pathological diagnosis, follow-up genetic diagnosis may provide follow-up help for LCNEC, and whether the follow-up prognosis can be predicted by related gene changes can be explored. In the era of precise treatment, it can improve the efficacy of pulmonary LCENC and prolong the survival period of patients. LCNEC patients also need more studies to make further progress in future treatment.
